# Evaluation of acetamiprid and azoxystrobin residues and their hormonal disrupting effects on male rats using liquid chromatography-tandem mass spectrometry

**DOI:** 10.1371/journal.pone.0259383

**Published:** 2021-12-02

**Authors:** Ekramy Halawa, Lamia Ryad, Nahla S. El-Shenawy, Rasha A. Al-Eisa, Heba N. Gad EL-Hak

**Affiliations:** 1 Agricultural Research Center, Central Lab of Residue Analysis of Pesticides and Heavy Metals in Food, Ismailia, Egypt; 2 Zoology Department, Faculty of Science, Suez Canal University, Ismailia, Egypt; 3 Biology Department, College of Sciences, Taif University, Taif, Saudi Arabia; Tianjin University of Traditional Chinese Medicine, CHINA

## Abstract

Endocrine-disrupting compounds as pesticides affect the hormonal balance, and this can result in several diseases. Therefore, the analysis of representative hormones with acetamiprid (AC) and azoxystrobin (AZ) was a good strategy for the investigation of the endocrine-disrupting activity of pesticides. Hence, a sensitive and rapid analytical method using liquid chromatography-tandem mass spectrometry (LC-MS/MS) was developed. The method was validated for the analysis of AC, AZ, estriol, estrone, progesterone, and testosterone in the serum, testis, and liver of rats. The correlation between the residues of pesticides and the disturbance of the endocrine system was evaluated. The different mass parameters, mobile phase types, analytical columns, injection volumes, and extraction solvents were compared to get the lowest limit of detection of the studied compounds. The detection limits of AC, AZ, estriol, estrone, progesterone, and testosterone were 0.05, 0.05, 1.0, 10, and 1.0 ng/ml, respectively. The method developed was applied to evaluate the changes in these hormones induced by the duration of exposure to AC and AZ in rat testis and serum. The hormones level in rat serum and testis had a significant decrease as they were oral gavage treated with different high concentrations of studied pesticides. Both pesticides were distributed in the body of rats by the multi-compartment model (liver, testis, and serum).

## Introduction

Endocrine-disrupting compounds (EDCs) are responsible for many disorders of the human hormone system. This alteration can cause a lot of disorders, like decreasing fertility, malformations in the new birth, and change in the sex ratio of humans [1, 2]. Also, EDCs are molecules in our environment, food, and consumer items that interfere with hormone biosynthesis, metabolism, or function, resulting in disruption of normal homeostatic regulation and reproduction [3, 4]. The enzymatic activities responsible for the balance of androgen and estrogen hormones may be disrupted by the effect of EDCs, leading to hormonal imbalance and different health disorders [4, 5]. EDCs work through a variety of mechanisms, including estrogenic, antiandrogenic, thyroid, peroxisome proliferator-activated receptor, retinoid, and other nuclear receptor effects. Steroidogenic enzymes, neurotransmitter receptors, and systems are all examples of these receptors. Many additional mechanisms are highly conserved in both animals and people and can be recreated *in vivo* and *in vitro* [2, 6–8].

There are nine putative mechanisms for EDCs in humans and/or in wildlife animals [9]. Only one mechanism out of these nine was confirmed, in which EDCs acted like hormones and bound to the hormone receptors leading to activation of their signaling pathway [10]. Each year, the number of potential EDC compounds increases, i.e. dioxins, polybromodiphenyl ethers, phthalates, parabens, alkylphenols, pesticides [6, 11].

Most regulatory tests available to study EDCs toxicity have been developed on rats as an animal model for endocrine screening [12]. Therefore, a validated sensitive analytical method has studied the effect of EDCs on rats’ hormonal systems [13]. However, the determination of pesticides and hormones in biological matrices such as serum or tissues is challenging as it needs a low limit of quantitation (LOQ). The LOQ is the lowest concentration at which the analyte can be quantitated at defined levels for imprecision and accuracy. Many difficulties were considered, such as the small sample amount and the effects of interfering compounds like fats and protein [6].

Most of the previous analytical methods for the determination of EDCs in serum or tissue were developed for a single-family of chemicals [14–16]. Moreover, estrogenic hormone concentrations after exposure to EDCs were determined [17, 18]. Analytical techniques typically depend on solid-phase extraction (SPE) or liquid-liquid extraction (LLE), accompanied by gas chromatography with mass spectrometry (GC–MS) or liquid chromatography combined with tandem mass spectrometry (LC-MS/MS) [19]. GC–MS is less popular now than LC-MS/MS because it needs a step of derivatization [20].

Acetamiprid (AC; neonicotinoids, insecticide) and azoxystrobin (AZ; fungicide) have been reported as low-risk pesticides due to their low toxicity [21–23]. All AC act on the insect central nervous system as agonists of the postsynaptic nicotinic acetylcholine receptors [24]. On the other hand, AZ was introduced as a pesticide class called ß-methoxyacrylates, and its biochemical mode of action is inhibition of electron transport [25]. Because of the excessive use of these pesticides, residues usually happen. Therefore, the international and national food/health authorities set a maximum residue limit (MRL) to regulate the correct use of pesticides to ensure food safety. Use MRL as the worst-case scenario could overestimate the potential risk of pesticide residues dietary intakes to humans. Moreover, it is well known that residue levels can be affected by food processing [26]. The provisions amend Annexes II and III for regulation (EC) No. 396/2005, establishing the maximum residue levels applicable to certain pesticides in or on certain products.

In this context, this study aimed to develop a fast, sensitive, and validated LC-MS/MS method for two EDCs (AC and AZ) and four hormones. These were carried out by comparing the different extracted solvents, high-performance liquid chromatography (HPLC) columns, and mobile phases. The validated method was used for the determination of the changes in testosterone and progesterone levels in the liver, testis, and serum due to the intake of different doses of AC and AZ.

## Materials and methods

### Chemicals and reagents

Standard reference materials of AC, AZ, estriol, estrone, progesterone, and testosterone were obtained from Dr. Ehrenstorfer GmbH (Augsburg, Germany, CHEM Europe. com). All reference standard materials were purity > 94%. Acetonitrile (ACN) and methanol of LC-MS/MS grade were purchased from Sigma-Aldrich (USA). Chemicals such as formic acid (FA) and ammonium formate (AF) were obtained from Sigma-Aldrich. De-ionized water (DIW) was available from a Millipore water purification system.

### Separation and detection conditions

Reversed-phase liquid chromatography was performed using an Agilent Poreshel 120 EC-C18 50 X 3 mm, 2.7 μm (Agilent Technologies, Santa Clara, CA, USA) column. The Exion LC system (Sciex/MDS SCIEX, Concord, Canada) coupled with a triple-stage quadrupole/linear ion trap mass spectrometer, model 6500^+^ Q TRAP (Sciex/MDS SCIEX, Concord, Canada) was used. The column temperature was 40°C, and the injection volume was 5μL. The separation was done using gradient elution by using the mobile phase (bottle A, 1% FA in water; and bottle B, methanol) with a flowrate of 0.3 mL/min. The following gradient elution program was utilized for chromatographic separation: 0 min (100% A), 1–3 min (0% A), and 3.5–5 min (100% A). The mass analysis was performed with multi reaction monitoring modes (MRM) by electrospray ionization in positive ion mode. The following parameters were used: temperature, 450° C; curtain gas, 25 psi; collision gas, medium; ion spray voltage, 5000 V; ion source gas 1, and 40 psi; and ion source gas 2, and 40 psi.

The positive mode for estrogenic compounds was mentioned before [27, 28]. However, for AC and AZ the positive mode is more sensitive than the negative mode. Thus, we preferred to apply the positive mode to analyze hormones and pesticides in one method together.

### Standard preparation

Individual standard stock solutions of 500 μg/ml were prepared in methanol for every compound. Mixture working solutions for spiking of concentrations 10, 50, and 250 ng/ml were prepared in acetonitrile according to the LOQ for each compound (AC, AZ, estriol, estrone, progesterone, and testosterone i.e. 0.05, 0.05, 50, 10, 1.0, and 0.1 ng/ml, respectively) (Table 1). Matrix matched calibration solutions of concentrations of 0.1, 0.5, 1.0, 2.5, 5, and 10 ng/mL were prepared for the liver extract in acetonitrile. Standard solutions of AZ (50 mg/ml) and AC (3000 μg/ml) for dosing purposes were prepared in water weekly.

**Table 1:** Quantification limit using LC-MS/MS for acetamiprid, azoxystrobin, estriol, estrone, progesterone, and testosterone in rat liver.

	Acetamiprid	Azoxystrobin	Estriol	Estrone	Progesterone	Testosterone
LOQ (ng/g)	0.05	0.05	50	10	1	1
**Mean Recovery LOQ**	99%	107%	92%	66%	82%	72%
**RSD LOQ**	15%	10%	8%	20%	9%	12%
**Mean Recovery 5 LOQ**	85%	88%	87%	76%	72%	93%
**RSD 5 LOQ**	9%	8%	10%	13%	9%	11%
**Mean Recovery 25 LOQ**	71%	74%	67%	79%	91%	80%
**RSD 25 LOQ**	12%	13%	10%	12%	11%	10%

RSD is the relative standard deviation. (n = 5).

### Extraction method

The extraction procedure was developed for testis, liver, and serum. The 0.5 g of testis and liver samples were transferred to a 50 ml centrifuge tube. Then, 10 ml of ethyl acetate (EA) was added and shaken for 10 min at 500 rpm with 2 steel balls to grind this small amount of tissue. The samples were centrifuged at 4500 rpm for 5 min. After that, all aliquots of the EA (10 ml) were transferred into a tube and evaporated under a nitrogen stream at 40°C. The samples were reconstituted with 1 ml ACN and injected into LC-MS/MS. The dilution factor was calculated as follows:

$$Dilution\;factor\;for\;liver\;or\;testis=\frac{10\;ml\;EA}{0.5\;g\;sample}\times \frac{1\;ml\;ACN}{10\;ml\;EA}=2$$ Eq 1

For serum, the extraction was performed by the liquid-liquid method via adding 500 μL of ethyl acetate to 100 μL of serum and sonication for 10 min. The mixture was shaken on a shaker for 1 min at 500 and centrifuged at 4500 rpm for 5 min. Then, the supernatant was collected by an acrodisc syringe filter (0.45 μm) and injected into LC-MS/MS. The sample was calculated with a dilution factor of 5 (Eq 2).


$$Dilution\;factor\;for\;serum=\frac{500\;\mu L\;EA}{100\;\mu L\;sample}=5$$ Eq 2

### Validation

**Fig 1:**
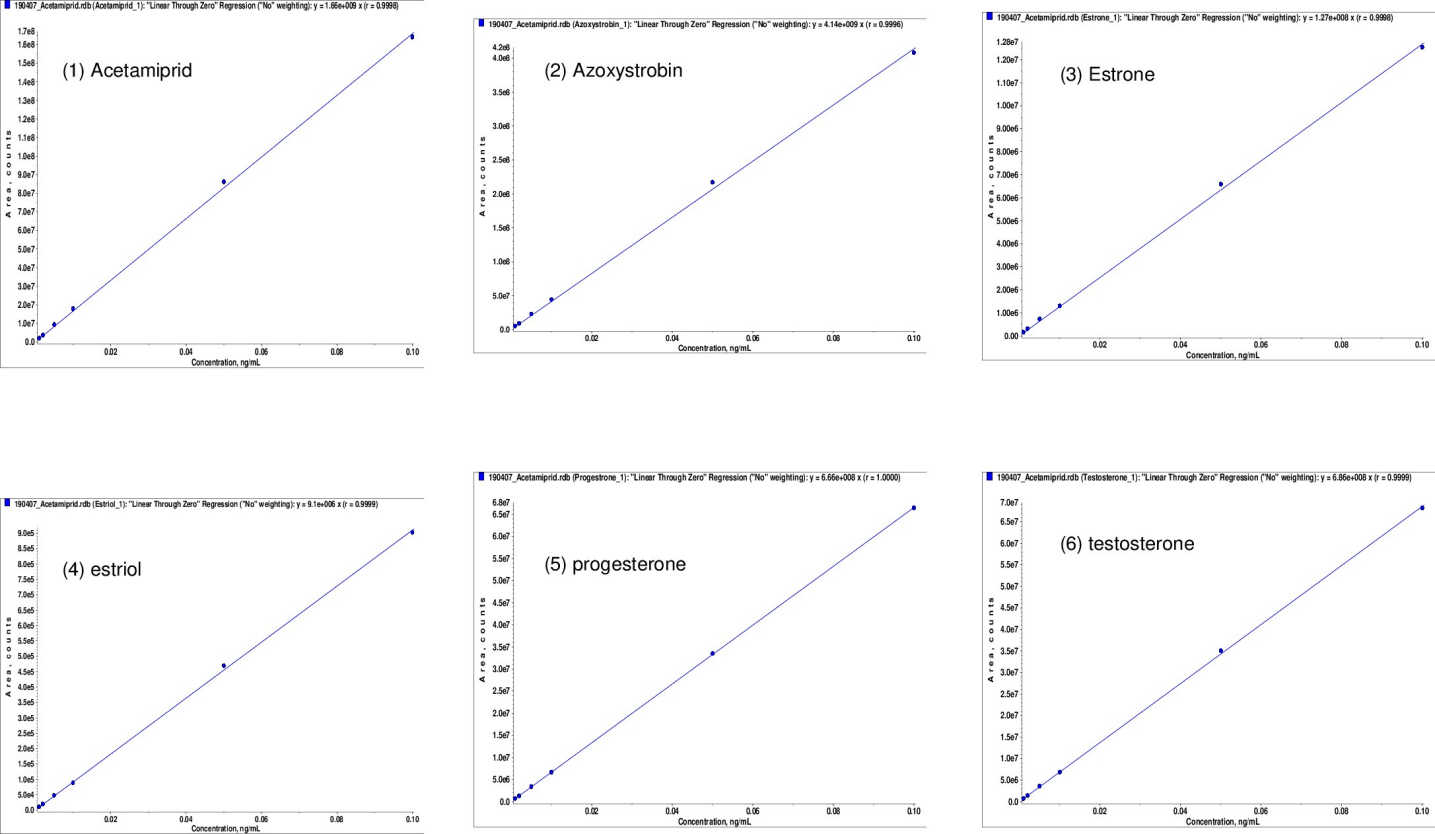
Linearity coefficients of calibration curves for all six compounds (acetamiprid, azoxystrobin, estrone, estriol, progesterone, and testosterone).

The method was validated for recovery, linearity (Fig 1), accuracy, and precision according to the guidelines for Analytical Procedures and Methods Validation for Drugs and Biologics by the United States Food and Drug Administration [29]. The limits of detection and quantification (LOD and LOQ) were determined by an analytic spiked level that produced a chromatographic peak signal of 3- and 10-times the background noise, respectively [30]. The method procedure was further evaluated using spike recoveries at three different concentrations (1, 5, and 25 μl LOQ) in the liver, and accuracy was confirmed by repeatability [29].

### Experimental design and sample collection

The experiments were conducted following the European Directive 2010-63-EU and approved by the local Animal Care and Use Committee (NO. 2012–5012041) [31]. Rats were provided by the National Research Centre’s (NRC) Animal Breeding House, Dokki, Giza, Egypt. All efforts were made to minimize the suffering and the number of animals used. Fifty-six adult male Wister rats weighing 140–160 g at age 60–80 days were used. They were kept together under observation for two weeks before the beginning of the experiment for acclimation under standardized conditions. The rats were housed in metal cages at a 24 ± 3°C temperature with normal light conditions (12 h light/dark cycle) and received food and water according to *ad libitum* feeding practice [32].

This experimental study was performed with the confirmation of the local ethics committee on the use and care of animals in experiments at the Faculty of Science, Suez Canal University, Egypt (permit number: REC57/2021).

**Fig 2:**
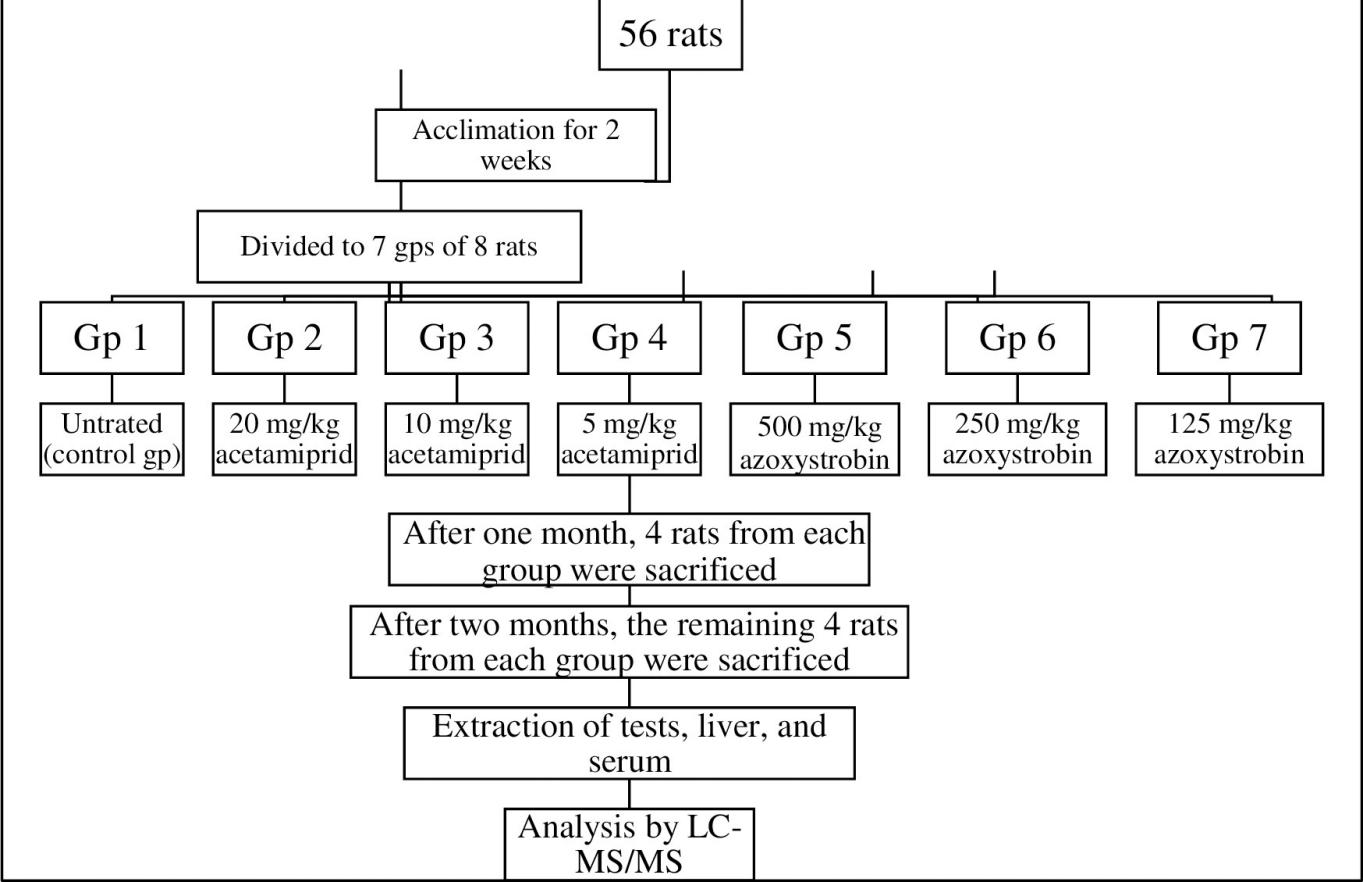
Experiment design to study the toxicity of acetamiprid and azoxystrobin on rats.

The LD_50_ of AC was taken as 200 mg/Kg [31, 33], and for AZ was 5000 mg/Kg according to EPA [25]. After 2 weeks of acclimation, animals were randomly divided into seven groups each of eight rats: Group 1 served as control. Groups 2, 3, and 4 were treated daily with 1/10-, 1/20-, and 1/40-LD_50_ of AC (20, 10, and 5 mg/kg b wt., respectively) by oral gavage. Groups 5, 6, and 7 were given 1/10-, 1/20-, and 1/40-LD_50_ of AZ (500, 250, and 125 mg/kg b wt., respectively) [34, 35] (Fig 2).

The volume for each dose was calculated depending on rat weight (g) as illustrated in Eq 3. Four rats from each group were anesthetized after a month of pesticide treatment. The rest of the animals in each group were sacrificed under anesthesia after two months. The mix of ketamine: xylazine at a dose of 80–100 mg/kg and 5–10 mg/kg (X) IP was used as an anesthetic agent, respectively. Different estrogenic hormones and residual pesticides were determined by using LC-MS/MS in liver, tissue, and serum samples.


$$Injection\;volume\;(ml)=\frac{rat\;weight\;(gm)\;X\;needed\;dose\;(mg/kg)}{used\;concentration\;of\;pesticide\;(ug/ml)}$$ Eq 3

### Statistical analysis

Data were presented as mean ± standard error (S.E.). Results were analyzed using analysis of variance (ANOVA; Two-way), followed by post hoc to compare between groups with SPSS for Windows version 20.0. The level was regarded to be at a *P*-value ≤ 0.05.

## Results and discussion

### LC-MS/MS analysis

**Fig 3:**
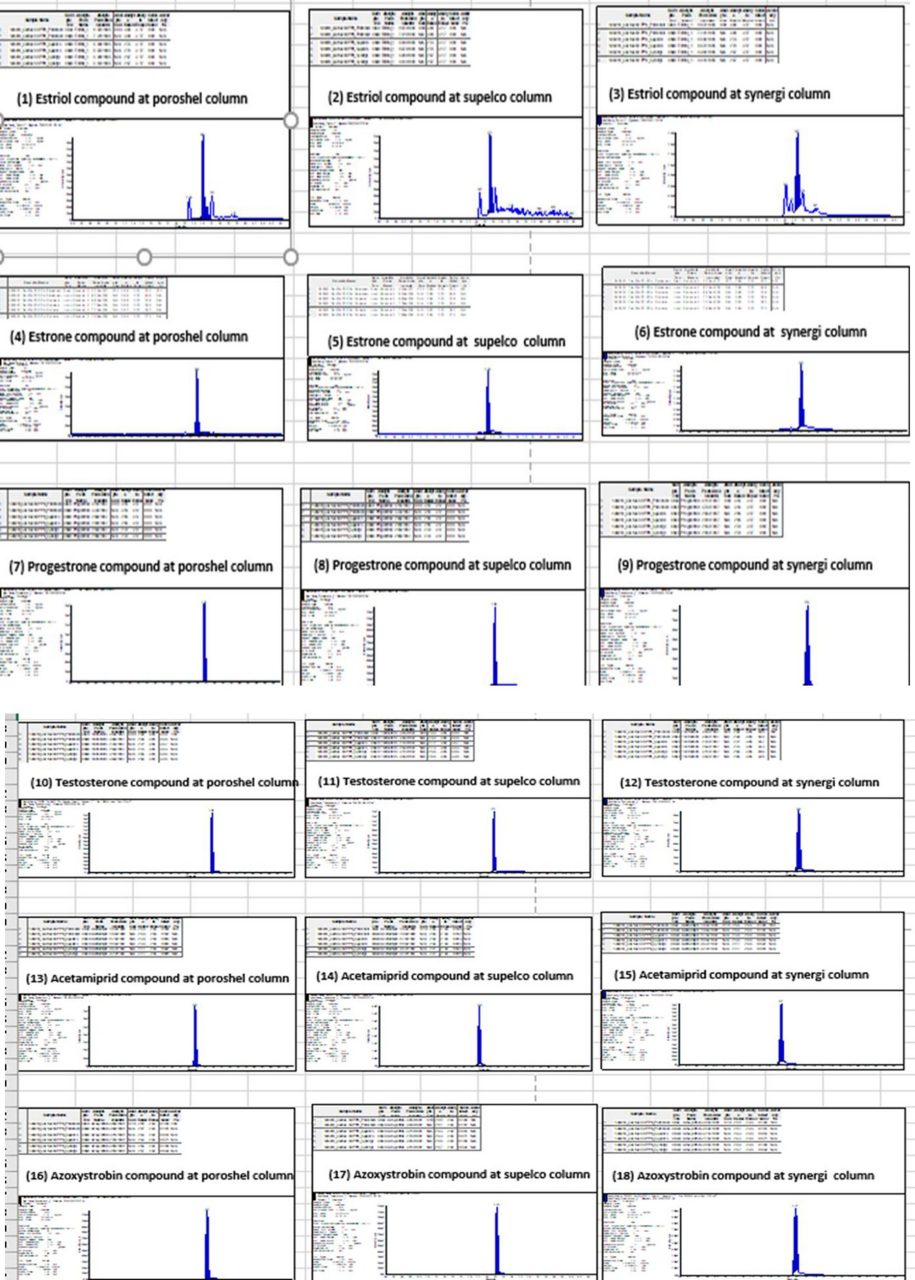
A. Column comparison (poroshel, supelco, and synergi) with three compounds (estriol, estrone, and progesterone). HPLC columns comparison (n = 5). Poreshel 120 EC-C18 50X 3 mm, 2.7 μm (Agilent), Titan C18 50 X 2.1 mm, 1.9 μm (Supelco), and Synergi Fusion 50 X 2 mm, 4 μm (Phenomenex). B. Column comparison (poroshel, supelco, and synergi) with three compounds (testosterone, acetamiprid, and azoxystrobin). HPLC columns comparison (n = 5). Poreshel 120 EC-C18 50X 3 mm, 2.7 μm (Agilent), Titan C18 50 X 2.1 mm, 1.9 μm (Supelco), and Synergi Fusion 50 X 2 mm, 4 μm (Phenomenex).

Three columns were tested for determination of AC, AZ, estriol, estrone, progesterone, and testosterone: Synergi Fusion 50 X 2 mm, 4 μm (Phenomenex), Titan C18 50 X 2.1 mm, 1.9 μm (Supelco), and Poreshel 120 EC-C18 50 X 3 mm, 2.7 μm (Agilent). More sharp peaks and selectivity were obtained in the case of Poreshel and Titan because of the smaller particle size than in Synergi Fusion (Fig 3A and 3B). As a result, Poreshel was used because of its higher sensitivity to progesterone and testosterone, estriol, and estrone, as well as because it is characterized by low column pressure as compared with the Titan column.

Two organic mobile phase compositions were tested: acetonitrile and methanol. However, there is no difference either in the peak shape or sensitivity of the target compounds. Moreover, three aqueous mobile phases were tested for the determination of AC, AZ progesterone, and testosterone; 0.1% FA, 5 mM ammonium formate + 0.1% FA (pH = 2.7), and 10 mM ammonium format buffer pH 4. AZ and testosterone have the same sensitivity in all mobile phases. However, the sensitivity of AC was duplicated in the case of using a 10 mM ammonium format buffer. Estriol, estrone, and progesterone had double sensitivity in the case of using mobile phase 0.1 FA compared with using buffer solutions. The 0.1% FA was used to enhance the low sensitivity of these compounds.

**Fig 4:**
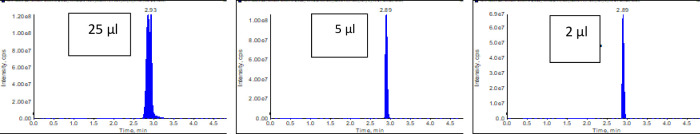
Extracted ion chromatogram of azoxystrobin 50 ppb, injection volume of 2, 5, and 25 μL. a 5 μL injection volume was chosen to achieve maximum detection and quantification limitations.

To get the lowest possible LOQ in the case of testosterone, estriol, and estrone, we attempted to increase the injection volume as much as possible. However, increasing the injection volume has its collateral bad effects on peak shape, the linearity of calibration, and the matrix effect. Three injection concentrations in the reference range of 0.5, 1, 2, 5, 10, and 1.0 ng/ml were tested using the volumes of 2, 5, and 25 μL. So, the response was determined for each volume of injection. In all cases, a linear response to the volume of injection was observed with no reduction in the consistency of chromatographic separation (Fig 4). Thus, a 5 μL injection volume was chosen to achieve maximum detection and quantification limitations.

**Fig 5:**
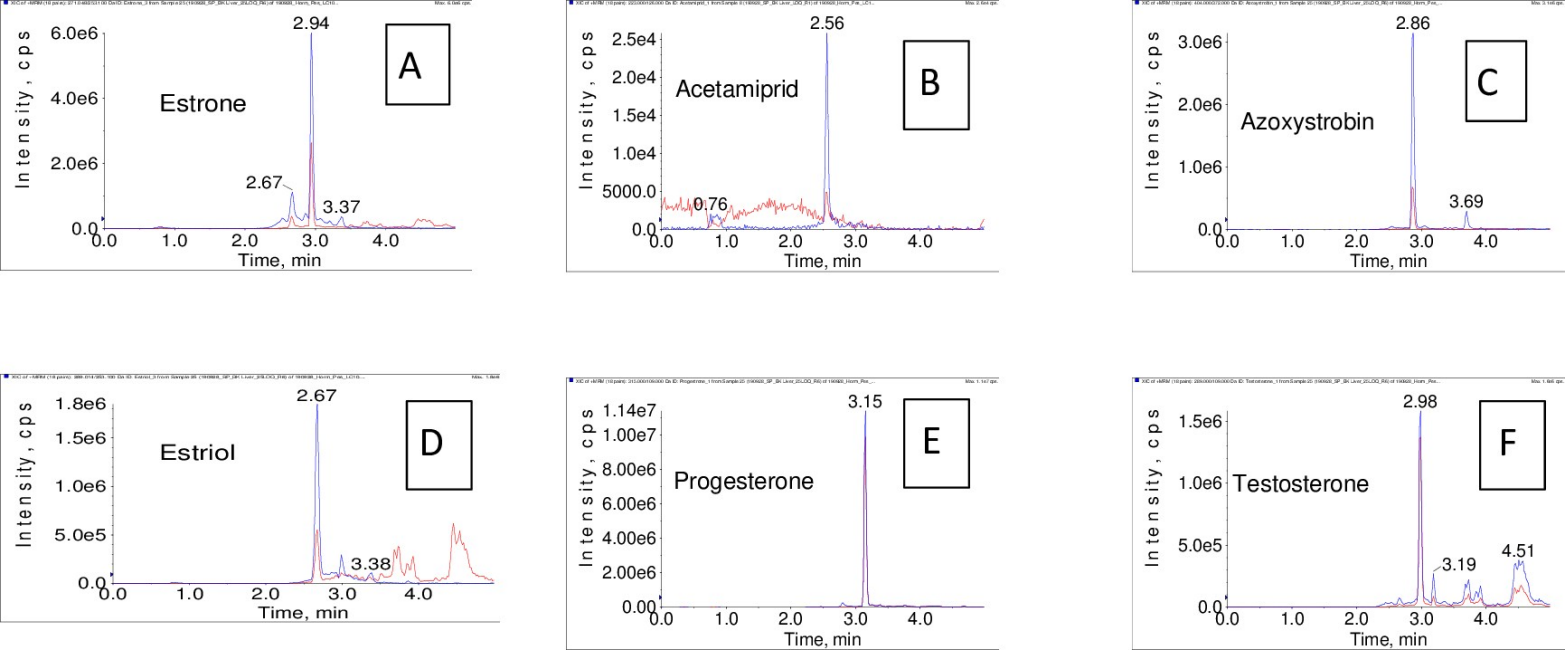
LC-MS/MS chromatograms of a spiked liver sample on LOQ level.

The U.S. Food and Drug Administration Guideline (FDA) for the Bio-analytical system verification and confirmation study approach [29, 30]. The full validation process in the liver tissue was performed for AC, AZ, estriol, estrone, progesterone, and testosterone (Table 1). LC-MS/MS chromatograms of a spiked liver sample at the LOQ level are presented in Fig 5.

Specific mass parameters that are set for each transition were designed for collision energy (CE), declustering potential (DP), entrance potential (EP), and collision exit potential (CXP). This was achieved by directly infusing veterinary medicines into the mass system in individual solutions. The transformations of the precursor/production and their related parameters are shown in Table 2.

**Table 2:** Mass parameters of parent and daughter ions of pesticides and hormones on LC-MS/MS (n = 5).

ID	Q1 (Dalton)	Q3 (Dalton)	T	DP	EP	CE	CXP
(Compound name)	(parent ionized molecule)	(fragment ionized molecule)	(dwell time)	(Declustering potential voltage)	(Entrance potential voltage)	(collision energy voltage)	(Exit potential voltage)
Acetamiprid-1	223	126	50	46	10	31	6
Acetamiprid-2	223	90	50	46	10	51	6
Azoxystrobin-1	404	372	50	50	10	20	10
Azoxystrobin-2	404	344	50	50	10	35	10
Estriol-1	289	107	50	100	10	23	6
Estriol-2	289	107	50	91	10	29	12
Estrone-1	271	253	50	89	10	19	14
Estrone-2	271	159	50	89	10	29	18
Progestrone-1	315	109	50	71	10	35	6
Progestrone-2	315	97	50	71	10	33	4
Testosterone-1	289	109	50	51	10	35	6
Testosterone-2	289	97	50	51	10	33	6

Where Q1and Q3 unit is Dalton, T time by millisecond, DP, EP, CE and CXP unit is millivolt.

#### Extraction optimization

For both liver and serum, three extraction solvents were compared, and the best solvent, in terms of high recovery and low matrix extraction was chosen (Table 3). The use of ethyl acetate and acetonitrile were reported before the extraction of hormones and pesticides from serum [36]. Moreover, the solvent mixture of ethyl acetate and hexane was used as reported before [37]. Therefore, acetonitrile, ethyl acetate, and ethyl acetate/hexane were used for the extraction comparison in the current study. The use of the LC-MS/MS instrument has become the instrumental technique of choice for the precise and reliable determination of trace compounds in complex food and feed materials. However, in routine LC-MS/MS analysis, the extraction procedures are most commonly important and applied.

**Table 3:** Extraction solvent comparison by using different solvents in the liver tissue (n = 5).

	Standard Matrix Recovery %			Average spike recovery %			RSD %		
	EA	EA/Hex	ACN	EA	EA/Hex	ACN	EA	EA/Hex	ACN
**Acetamiprid**	70	89	10	84	73	66	10	15	14
**Azoxystrobin**	84	95	75	80	69	84	11	10	8
**Estriol**	14	8	6	65	65	49	9	30	44
**Estrone**	21	14	13	81	64	83	7	19	13
**Progesterone**	68	74	57	80	81	73	11	20	16
**Testosterone**	12	18	12	85	51	53	20	23	54

EA = ethyl acetate, EA/Hex = ethyl acetate /hexane 1:1, ACN = acetonitrile, RSD = relative standard deviation.

Recent sample preparation protocols approach which extraction solvent is applicable for multiple analyses of various substance classes. Whereas the optimal extraction solvent efficiency properties were the high recovery of the target compounds (spiked on the blank liver) and low matrix effect (non-target substance). In the present study, the optimal solvent among compared solvents was ethyl acetate as the lowest relative standard deviation (RSD) in all compounds (AC, AZ, estriol, estrone, progesterone, and testosterone equal 10, 11, 9, 7, 11, and 20, respectively). Also, ethyl acetate gave a high accepted average spiked recovery results (AC, AZ, estriol, estrone, progesterone, and testosterone equal 84, 80, 65, 81, 80, and 85, respectively).

Although the solvent mixture of ethyl acetate and hexane gave a realistic matrix effect in most compounds, its RSD for estriol was 30% and spike recoveries for testosterone were 51%. The acetonitrile extraction solvent gave a high matrix effect and low recovery in the case of AC, estriol, and testosterone (Table 3). The RSD of testosterone was reported to be 54%. The same results were obtained in the case of serum, but with fewer differences between extraction solvents. This might be due to the low fat and protein content of serum samples compared to liver samples.

Moreover, the use of steel balls instead of ultraturax came with two benefits. the first is the best grinding efficiency compared to ultraturax, without wasting a small amount of the sample. It was not possible, practically, to use ultraturax with this small amount as it stuck in the machine. The use of steel balls was reported before in the determination of veterinary drug residues in milk powder [38], but this is the first time, to our knowledge, to be used in animal tissue analysis.

### Linearity coefficient of the calibration curve

Calibration curves prepared in neat solvents yielded *R*^*2*^ values of 0.994 to 1.00 in the concentration range of 0.5, 1, 2,5,10, and 50 ng/ml for all estrogen hormones and pesticides. Matrix-matched calibration curves for liver extract at fortified levels of 0.5, 1, 2.5, and 10 ng/ml displayed impressive *R*^*2*^ values above 0.999 for AC, progesterone, and testosterone. However, AZ, estriol, and estrone had *R*^*2*^ values of 0.998, 0.971, and 0.987, respectively, during the mentioned range of matrix-matched calibration (Fig 1). Matrix effect compensation was calculated for serum and testis.

### Selectivity

The liver was chosen as a representative sample of tissue in the validation because it is hard to obtain a blank testis sample, i.e. free of testosterone and progesterone. Moreover, the analysis of the blank was carried out. The comparison of the blank sample chromatogram with the corresponding spiked sample containing a known analytic concentration revealed the absence of interference from the studied substances at the time of retention of all analyses.

Similarly, optimized instrumental conditions ensured high selectivity of the proposed system. Fig 4 displays a chromatogram of a reinforced sample in MRM. It stands out from the injection of estrone and testosterone that there are interfering peaks of testosterone that appear in estrone transitions. With the poor HPLC separation of estrone and testosterone, the determination of estrone using this method is only possible if the sample is free of testosterone.

As a kind of impartiality, we found that when the testosterone compound was present, the estrone compound was found. Objectively, it may be due to the conversion of testosterone compound (parent with 289 Dalton) to estrone compound (parent with 271 Dalton) by losing water molecules (18 Dalton). Estrone was thought to have a much better MS/MS response in the negative ionization mode [39]. However, in the current approach, positive ionization transitions (271/253 and 271/159) of estrone had higher sensitivity than negative ionization transitions. Moreover, selecting the positive mode avoided losses in sensitivity for a positive/negative switching ionization method [40].

### Recoveries

As seen in Table 1, the recoveries of studied compounds varied between 66% and 107%, with an average of 81%. These results are comparable with previous studies dealing with the analysis of hormones and pesticides as well as in biological samples [41]. The minimum recoveries were 66% and 68% for estrone and estriol. A similar low recovery was obtained for these compounds previously [41].

### LOQ and LOD

Despite the short processing time for the proposed method, low LOQs and LODs were obtained for the tested compounds. The LOQ of AC and AZ was 0.05 ng/ml. However, it was reported in the literature that AC was determined with LOQs of 50 ng/ml in serum and liver [41, 42]. Regarding estrogenic hormones, low LOQs were introduced compared to the literature concerning the determination of hormones in tissue or serum. The LOQs of estriol, estrone, progesterone, and testosterone in the present study were 50, 10, 1.0, and 1.0 ng/ml, respectively (Table 1). However, in a previous study, the LOQs of estriol, estrone, and testosterone in liver samples were 300, 10, and 6 ng/g [43]. The presented LODs of AC, AZ estriol, estrone, progesterone, and testosterone were 0.02, 0.02, 20, 3, 0.03, and 0.03 ng/ml, respectively, calculating the signal to noise ratio 10 times.

### Repeatability and reproducibility

Repeatability or within-day reproducibility was represented by RSD percentages, and it was less than 15% in all compounds except for the level of LOQ of estrogen (20%). Inter-day reproducibility during five consecutive days ranged from 8.4% to 20%.

### Rat samples

Firstly, there were no significant reductions either in body weight or food consumption in all groups of the tested rats (Table 4), which might be because of the low used doses of both pesticides. The lowest observed adverse effect level (LOAEL) of AC and AZ during sub-chronic testing was 51.0 and 211 mg/kg/day [25, 44], respectively.

**Table 4:** Effect of acetamiprid and azoxystrobin on the body weight (g) of rats during the two months of exposure.

Groups	Zero time	First	Second week	Third week	Forth week	Fifth week	Sixth week	Seventh week	Eight week
**Control**	150.0 ± 0.01	180.2 ± 0.3	200 ± 3.2	225 ± 1.7	250 ± 1.5a	273 ± 0.8	295 ± 1.1	320 ± 2.1	348 ± 2.1 ^a,b^
**1/10-LD**_**50**_ **acetamiprid**		165 ± 2.0	185 ± 1.9	200 ± 2.8	220 ± 1.7 ^a^	230 ± 0.9	250 ± 2.3	270 ± 3.1	291 ± 3.2 ^a,b^
**1/20-LD**_**50**_ **acetamiprid**		170 ± 1.6	190 ± 2.4	208 ± 2.2	231 ± 2.7 ^a^	240 ± 0.6	261 ± 1.8	290 ± 2.2	296 ± 3.2 ^a,b^
**1/40-LD**_**50**_ **acetamiprid**		175 ± 1.4	194 ± 3.1	213 ± 3.1	237 ± 2.5 ^a^	250 ± 2.6	272 ± 2.1	295 ± 1.7	309 ± 1.5 ^a,b^
**1/10-LD**_**50**_ **azoxystrobin**		170 ± 2.1	183 ± 0.7	198 ± 2.2	217 ± 1.6 ^a^	233 ± 2.4	255 ± 3.1	271 ± 1.9	290 ± 2.3 ^a,b^
**1/20-LD**_**50**_ **azoxystrobin**		170 ± 1.8	190 ± 1.1	211 ± 3.2	232 ± 1.4 ^a^	242 ± 2.1	264 ± 2.3	293 ± 2.1	294 ± 3.4 ^a,b^
**1/40-LD**_**50**_ **azoxystrobin**		175 ± 1.7	195 ± 1.3	218 ± 2.4	235 ± 1.1 ^a^	255 ± 4.5	271 ± 2.3	297 ± 1.4	312 ± 1.8 ^a,b^

Data presented as mean ± S.E. (n = 4/group). Groups of 1/10-, 1/20-, and 1/40-LD_50_ of acetamiprid (20, 10, and 5 mg/kg b wt., respectively) by oral gavage. Groups of 1/10-, 1/20-, and 1/40- LD_50_ of azoxystrobin (500, 250, and 125 mg/kg b wt., respectively) (Türk et al. 2007; Kim et al. 2008, respectively). ^a^ Significant difference as compare to the first week, and ^b^ Significant difference as compare to the forth week.

**Fig 6:**
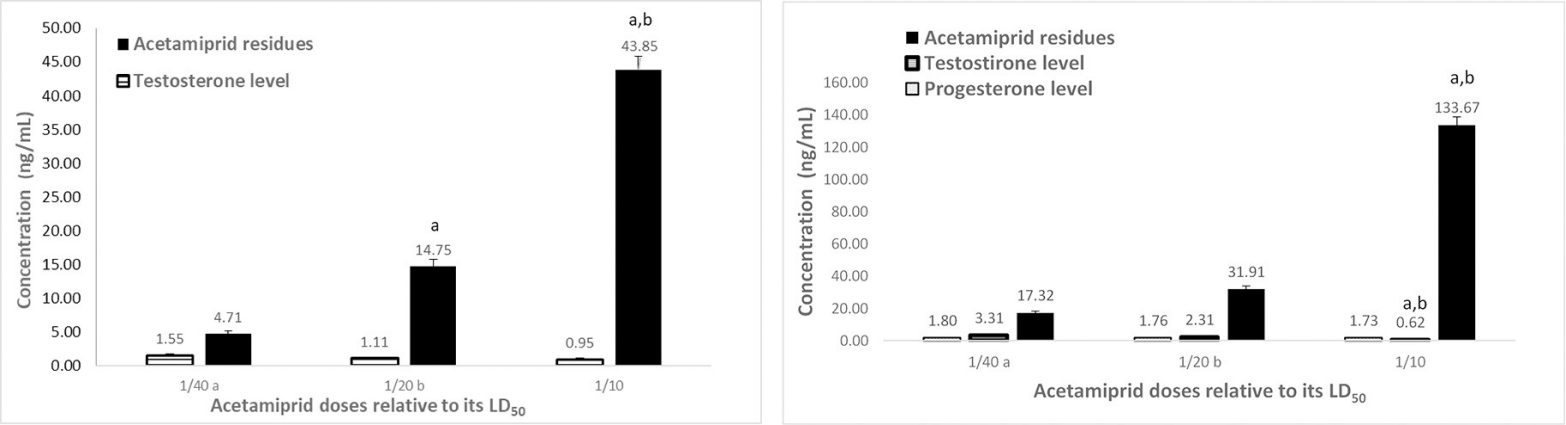
A. Serum testosterone levels and acetamiprid residues levels after a month of different acetamiprid doses treatment of the male rats. Data presented as mean S.E. ^a^ Significant difference as compared to 1/40 LD_50_, and ^b^ significant difference as compared to 1/20 of LD_50_ (n = 4, *P*≤ 0.05). B. Serum testosterone and progesterone levels as well as acetamiprid residues after 2 months of different acetamiprid doses treatment of male rats. Data presented as mean S.E. ^a^ Significant difference as compared to 1/40 LD_50_, and ^b^ significant difference as compared to 1/20 of LD_50_ (n = 4, *P*≤ 0.05).

**Fig 7:**
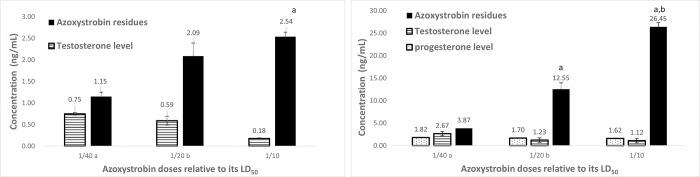
A. Serum testosterone levels and azoxystrobin residues after a month of different azoxystrobin doses treatment of male rats. Data presented as mean S.E. ^a^ Significant difference as compared to 1/40 LD_50_, and ^b^ significant difference as compared to 1/20 of LD_50_ (n = 4, *P*≤ 0.05). B. Serum testosterone and progesterone levels as well as azoxystrobin residues after 2 months of different azoxystrobin doses treatment on male rats. Data presented as mean S.E. ^a^ Significant difference as compared to 1/40 LD_50_, and ^b^ significant difference as compared to 1/20 of LD_50_ (n = 4, *P*≤ 0.05).

The levels of testosterone and progesterone significantly increased after two months as compared with the control at a month. Serum testosterone levels and AC residues after a month of treatment showed that the hormone levels decreased with increasing residues (Fig 6A). After two months of AC treatment, the testosterone level increased, the residue of pesticide was elevated, and the progesterone was detected (Fig 6B). The effects of AZ on the hormones and its residues in serum after two months of treatment are presented in Fig 7A and 7B.

**Fig 8:**
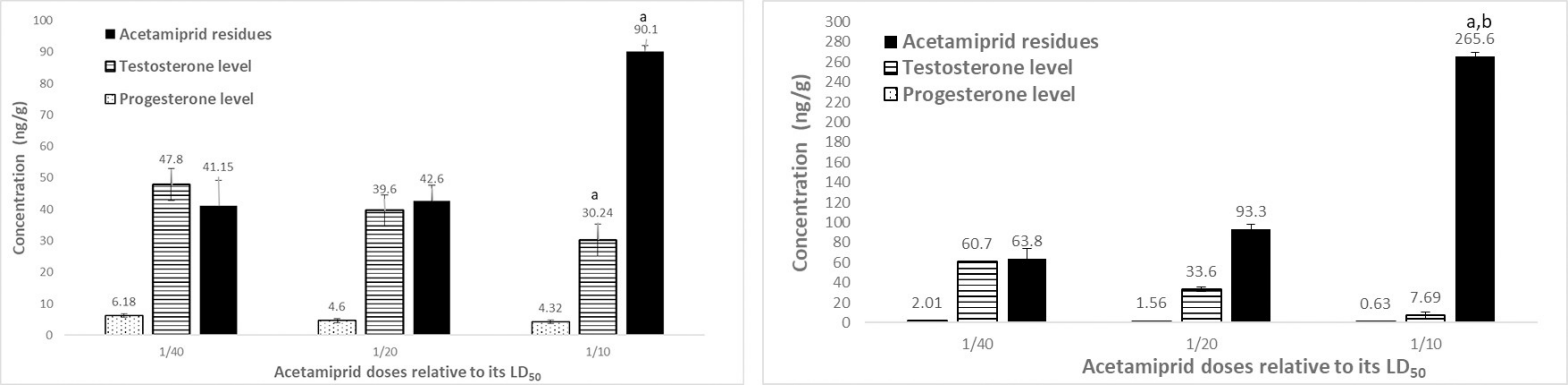
A. Testis testosterone and progesterone levels as well as acetamiprid residues after a month of different treatment of acetamiprid doses on male rats. Data presented as mean S.E. ^a^ Significant difference as compared to 1/40 LD_50_, and ^b^ significant difference as compared to 1/20 of LD_50_ (n = 4, *P*≤ 0.05). B. Testis testosterone and progesterone levels as well as acetamiprid residues after 2 months of different treatment of acetamiprid doses on male rats. Data presented as mean S.E. ^a^ Significant difference as compared to 1/40 LD_50_, and ^b^ significant difference as compared to 1/20 of LD_50_ (n = 4, *P*≤ 0.05).

**Fig 9:**
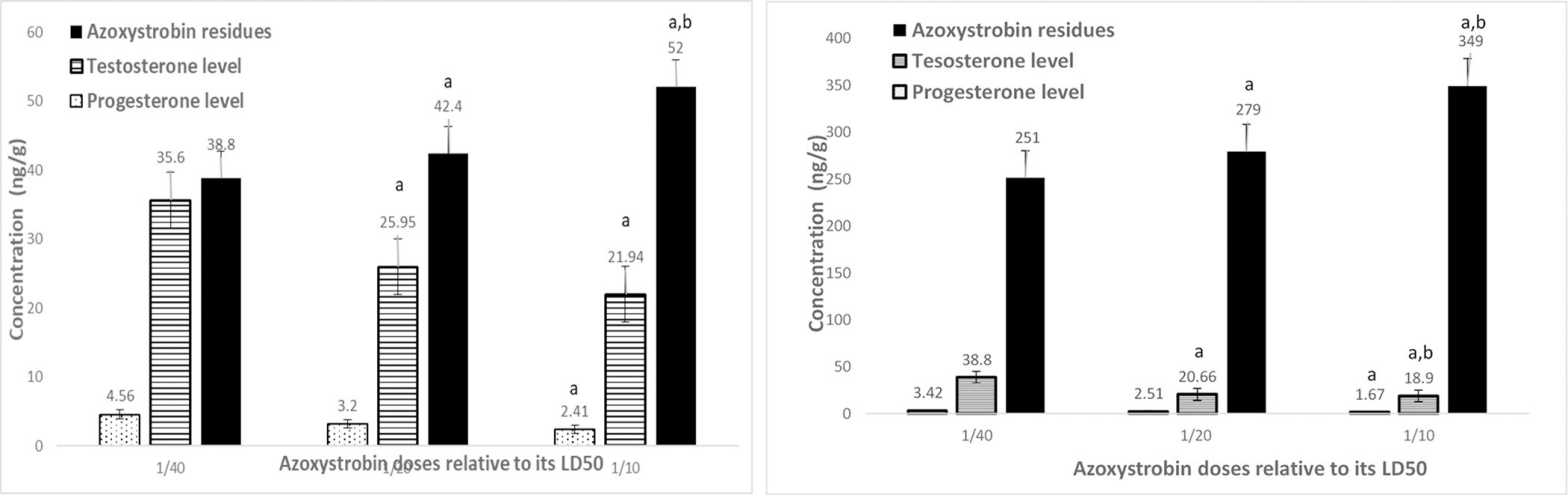
A. Testis testosterone and progesterone levels as well as azoxystrobin residues after a month treatment of different azoxystrobin doses on male rats. Data presented as mean S.E. ^a^ Significant difference as compared to 1/40 LD_50_, and ^b^ significant difference as compared to 1/20 of LD_50_ (n = 4, *P*≤ 0.05). B: Testis testosterone and progesterone levels as well as azoxystrobin residues after 2 months treatment of different azoxystrobin doses on male rats. Data presented as mean S.E. ^a^ Significant difference as compared to 1/40 LD_50_, and ^b^ significant difference as compared to 1/20 of LD_50_ (n = 4, *P*≤ 0.05).

The levels of testosterone, as well as progesterone, were detected in the testis after AC (Fig 8A and 8B) and AZ (Fig 9A and 9B) treatment for two months.

**Fig 10:**
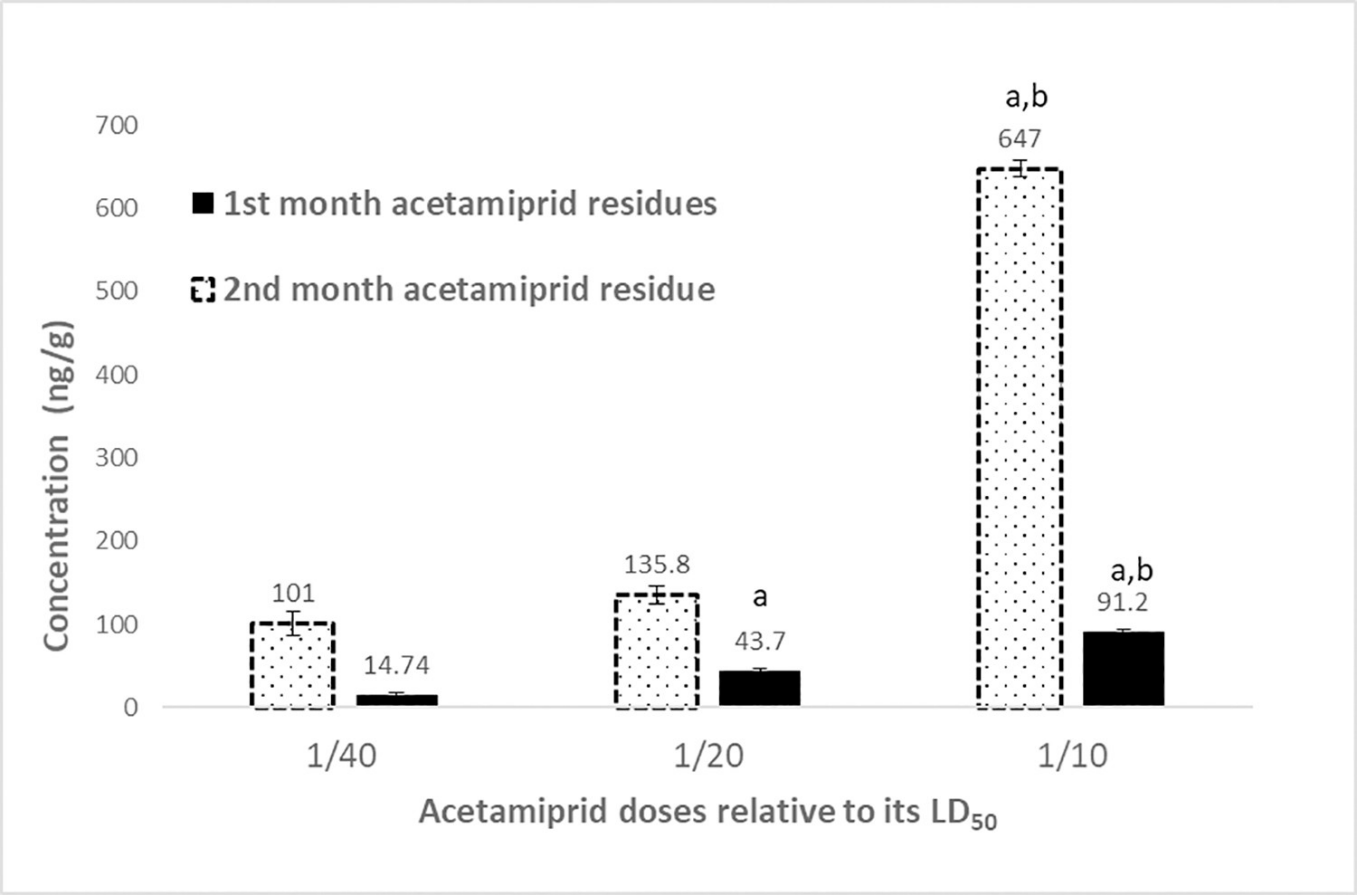
Liver acetamiprid residues after 1 and 2 months treatment of with different doses of acetamiprid on male rats. Data presented as mean S.E. ^a^ Significant difference as compared to 1/40 LD_50_, and ^b^ significant difference as compared to 1/20 of LD_50_ (n = 4, *P*≤ 0.05).

**Fig 11:**
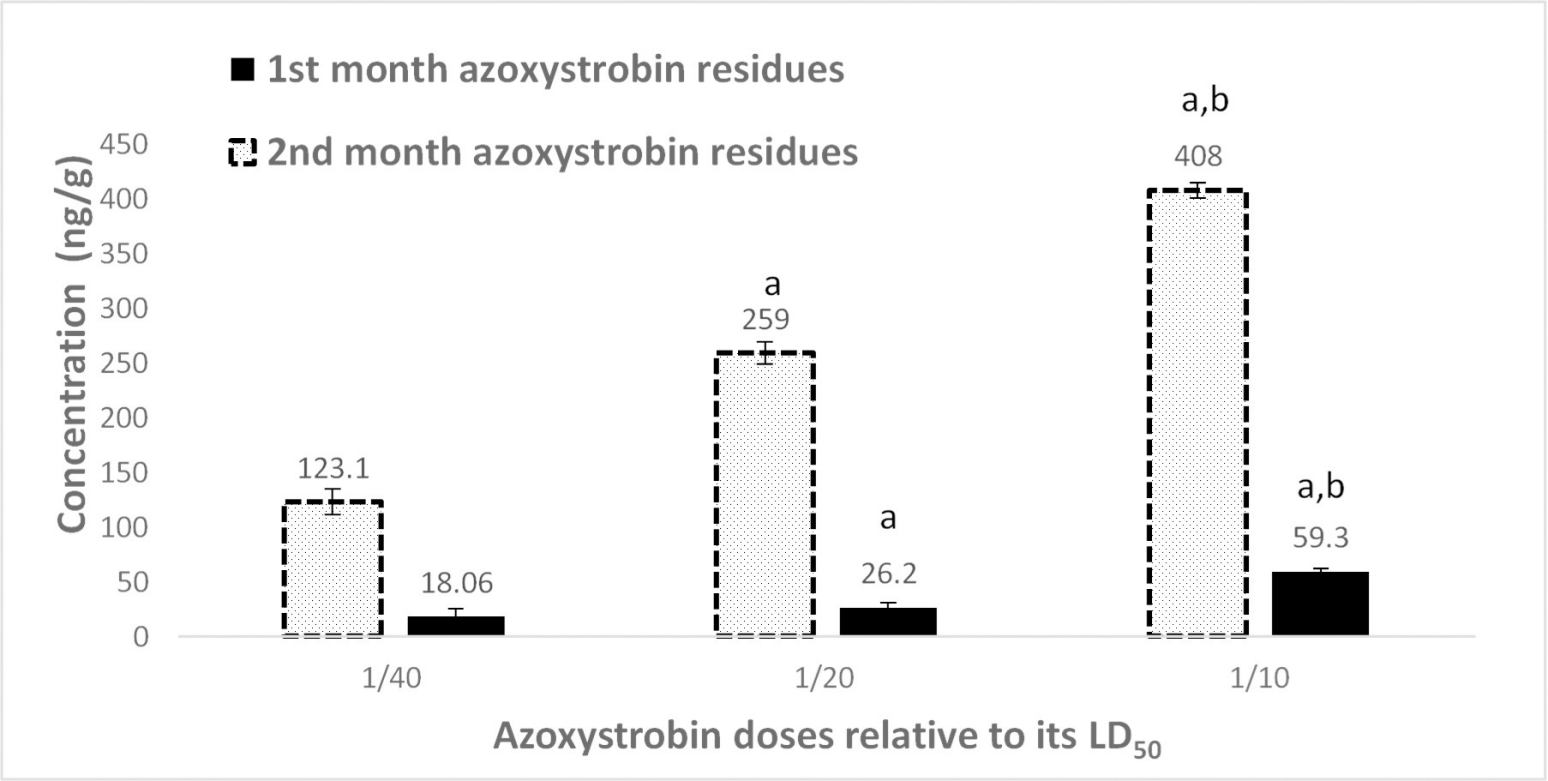
Liver azoxystrobin residues after 1 and 2 months treatment with different doses of azoxystrobin on male rats. Data presented as mean S.E. ^a^ Significant difference as compared to 1/40 LD_50_, and ^b^ significant (n = 4, *P*≤ 0.05).

Overall, the majority of AC doses were found to accumulate in the liver (Figs 10 and 11) rather than in the serum and testis, whereas in the case of AZ, it was equally distributed between the testis and liver with low residues determined in the serum. Although higher doses of AZ were used, AC had quite as many residues as that of AZ in rats’ organs. The high LD_50_ of AZ might be related to the mentioned low accumulation behavior in rats’ organs. This variation in residues of different compounds was reported in tracing of vinclozolin residue in rat testis in a former study of endocrine-disrupting of bisphenol, atrazine, methoxychlor, and vinclozolin [4, 45]. Moreover, the residues of both compounds were elevated with an increase in the duration of exposure to pesticides.

In the current study, the long period (30 to 60 days) led to a better understanding of the disrupting activity of AC and AZ. In contrast, the short dosing period of less than one month, which was reported in previous studies, was not enough to cause a significant alteration in hormones levels [46]. The testosterone level in serum in one and two months’ periods dropped from the normal level, i.e. the measured level of the control sample, of 4.15 and 6.23 ng/ml, respectively (Table 5), to about half, in the case of low concentrations of AC (1/20 and 1/40 of LD_50_). However, in the case of a high dose (1/10 of LD_50_) of AC, the testosterone levels were 0.95 and 0.62 ng/ml after the one-and two-month testing period, respectively.

**Table 5:** Mean values of testosterone and progesterone in control samples (n = 4/each month).

.	Progesterone level (ng/g)		Testosterone level (ng/g)	
	First month	Second month	First month	Second month
**Serum**	(not detected)	3.43 ± 0.09^a^	4.15 ± 0.50	6.23 ± 1.50 ^a^
**Testis**	6.98 ± 0.01	10.31 ± 0.21 ^a^	62 ± 5.50	90± 9.50 ^a^

The data presented as mean ± S.E.

^a^ Significant difference as compared to the data of a month (*P*≤ 0.05).

Moreover, the effect of AZ was quite more than AC in serum testosterone, while it fell to 0.18 ng/g after one month of AZ dosing instead of 0.95 ng/ml of AC. Similarly, the testosterone levels of testis declined by increasing either the dose and/or the duration of treatment with AC and AZ. The present results are in parallel with other studies [47, 48]; they investigated the different doses of AC on various modes of animals for a divergent duration and they found that the testosterone level was decreased. Since testosterone, the most common circulating androgen, is made from cholesterol [49], a drop in plasma testosterone may be due to a drop in plasma cholesterol levels. According to Eacker et al. [50], plasma cholesterol levels decrease dramatically in a dose-dependent fashion. The AC can also cause oxidative stress in Leydig cells, which can lead to a reduction in testosterone secretion [48].

On the other hand, it was reported in a previous study that the progesterone concentration in the serum of a 9–10 week male rat did not exceed 0.2 ng/g [46]. As a result, with a relatively high LOQ of 1 ng/ml, the progesterone concentration in the serum of the control samples in the first month could not be determined by the proposed method. However, in the second month, serum progesterone level was 3.43 ng/ml (Table 5). The AC and AZ had an equal effect on serum progesterone levels (Figs 4A and 5A). Moreover, the increase in the doses had not resulted in a further decrease in progesterone levels, with an average level of 1.7 to 1.8 ng/ml after all doses.

The determined progesterone concentration in the testis of control samples of one and two months were 6.98 and 10.31 ng/g (Table 5). As for AC, the decrease was sharper in the high dose and the long period of treatment, with 0.63 ng/g in 2 months using 1/10 LD_50_. Whereas, the decline in progesterone level because of AZ dosing was steady and proportional to the increase in period and/or dose (Fig 6A, 6B). There was an elevation in pesticides of liver tissue by increasing the dose and duration of the treatment (Figs 7A, 7B and 8, 9).

## Conclusion

A comprehensive analytical method was developed for simultaneous extraction and determination of two EDCs and some hormones in rat testis, liver, and serum. The developed method demonstrated good linearity, accuracy, and precision with good recoveries. Serum and testis hormone levels of rats decreased dramatically with increasing the concentrations of studied pesticides. AC was found to accumulate in the liver more than serum and testis, while AZ was distributed between the testis and liver with the low residue in serum. There was an elevation in the levels of pesticides in liver tissue by increasing the dose/duration of treatment. The testosterone level decreased by increasing either the dose and/or the duration of treatment of AC and AZ. There was a correlation between the residues of pesticides and the disturbance of the endocrine system. More studies will be conducted to evaluate the toxicity of these two pesticides on histology and biochemical of the testis and liver of rats.

## Supporting information

S1 Data (XLSX)

S2 Data (XLSX)

S1 File (DOCX)
