# Supplementary material for: Evaluation of acetamiprid and azoxystrobin residues and their hormonal disrupting effects on male rats using liquid chromatography-tandem mass spectrometry
Source: PLoS One. 2021 Dec 2;16(12):e0259383. doi: 10.1371/journal.pone.0259383 (PMC8638893; doi:10.1371/journal.pone.0259383)
Supplement: S1 File — (DOCX) [file pone.0259383.s003.docx]

Matrix match calibration curves were prepared in liver only as the liver could to obtain easily by a large amount than serum and testis. However another technique (standard in matrix) to calculate the serum and testis samples concentration was used. (we put them all in supplemented data file)

**Fig. 1: AC calibration curve**

**Fig. 2: AZ calibration curve**

**Fig. 3: Progesterone calibration curve**

**Fig. 4: Testosterone calibration curve**

**Fig. 5: Estrone calibration curve**

**Fig. 6: Estriol calibration curve**
